# Heat-killed *Limosilactobacillus reuteri* ATCC PTA 6475 prevents bone loss in ovariectomized mice: A preliminary study

**DOI:** 10.1371/journal.pone.0304358

**Published:** 2024-05-31

**Authors:** Jaqueline Lemes Ribeiro, Thaís Aguiar Santos, Maíra Terra Garcia, Bruna Fernandes do Carmo Carvalho, Juan Emmanuel Costa Sant’Ana Esteves, Renata Mendonça Moraes, Ana Lia Anbinder

**Affiliations:** 1 Department of Bioscience and Oral Diagnosis, Institute of Science and Technology of São José dos Campos (São Paulo State University–Unesp), São José dos Campos, São Paulo, Brazil; 2 Private Practice, São José dos Campos, São Paulo, Brazil; University of Vermont College of Medicine, UNITED STATES

## Abstract

Osteoporosis is an important health problem that occurs due to an imbalance between bone formation and resorption. Hormonal deficiency post-menopause is a significant risk factor. The probiotic *Limosilactobacillus reuteri* has been reported to prevent ovariectomy (Ovx)-induced bone loss in mice and reduce bone loss in postmenopausal women. Despite the numerous health benefits of probiotics, as they are live bacteria, the administration is not risk-free for certain groups (e.g., neonates and immunosuppressed patients). We evaluated the effects of *L*. *reuteri* (ATCC PTA 6475) and its heat-killed (postbiotic) form on Ovx-induced bone loss. Adult female mice (BALB/c) were randomly divided into four groups: group C—control (sham); group OVX-C—Ovx; group OVX-POS—Ovx + heat-killed probiotic; group OVX-PRO—Ovx + probiotic. *L*. *reuteri* or the postbiotic was administered to the groups (1.3x10^9^ CFU/day) by gavage. Bacterial morphology after heat treatment was accessed by scanning electron microscopy (SEM). The treatment started one week after Ovx and lasted 28 days (4 weeks). The animals were euthanized at the end of the treatment period. Bone microarchitecture and ileum *Occludin* and pro-inflammatory cytokines gene expression were evaluated by computed microtomography and qPCR techniques, respectively. The Ovx groups had lower percentage of bone volume (BV/TV) and number of bone trabeculae as well as greater total porosity compared to the control group. Treatment with live and heat-killed *L*. *reuteri* resulted in higher BV/TV and trabecular thickness than the Ovx group. The heat treatment caused some cell surface disruptions, but its structure resembled that of the live probiotic in SEM analysis. There were no statistical differences in *Occludin*, *Il-6* and *Tnf-α* gene expression. Both viable and heat-killed *L*. *reuteri* prevented bone loss on ovariectomized mice, independently of gut *Occludin* and intestinal *Il-6* and *Tnf-α* gene expression.

## Introduction

Osteoporosis is a systemic disease characterized by reduced bone mass and microarchitectural degradation, leading to bone fragility and an increased risk of fracture [1]. It represents a significant public health problem as it is estimated that over half of the individuals over 50 in the United States have osteoporosis or osteopenia [2]. One in three women and one out of five men over 50 will experience an osteoporosis-related fracture [3]. Postmenopausal osteoporosis is the most common due to reduced hormone production [4]. During estrogen deficiency, there is an increase in the production of pro-inflammatory and pro-osteoclastogenic cytokines such as RANKL, tumor necrosis factor (TNF)-α [5] and interleukin (IL)-17 [6]. Additionally, there is a decrease in the production of anti-inflammatory cytokines such as IL-10, IL-4, interferon (IFN)-γ, as well as osteoprotegerin, resulting in increased osteoclastogenesis and subsequent bone resorption [6].

“Osteomicrobiology” is a research field that focuses on the role of the microbiota in host health and bone diseases [7]. Both human and animal studies have made it clear that the gut microbiota is essential for the overall health of the host playing a critical role in metabolism (e.g., production of hormones, such as dopamine and serotonin), nutrition (e.g., minerals absorption), pathogen resistance (e.g., intestinal barrier), and the immune system [8, 9]. The intercellular space of the intestinal epithelium is sealed by tight junctions (TJs) composed of complex protein structures, such as junctional adhesion molecules, zonula occludens proteins, claudin, and occludin. TJs are dynamic structures that adapt to external stimuli, such as food residues, commensal and pathogenic bacteria [10]. Estrogen activates a series of pathways essential for maintaining TJs, thus increasing the transepithelial electrical resistance in the barrier, preventing the entry of pathogens. Therefore, estrogen deficiency weakens the intestinal epithelial barrier, allowing the invasion of antigens and bacteria. This invasion initiates an inflammatory immune response associated with postmenopausal bone loss [11].

Numerous therapies have been developed for the treatment of osteoporosis ranging from non-pharmacological measures (e.g., healthy lifestyle habits) to pharmacological treatments. These are recommended for patients at an increased risk of fracture and include medications such as bisphosphonates, selective estrogen receptor modulators, estrogen replacement therapy, and anabolic and antiresorptive drugs [12]. However, pharmacological therapy is associated with various adverse effects, including gastrointestinal reactions, osteonecrosis of the jaw [12], an increased risk of thromboembolic events, myocardial infarction, and breast cancer. These side effects have led to exploring new preventive and therapeutic possibilities, such as probiotics [13].

Probiotics are live, non-pathogenic microorganisms that, when administered in adequate amounts, offer benefits to the health of the host [14]. The consumption of probiotics has been associated with several advantages, including the maintenance of intestinal barrier function and strengthened immune system, which contribute to an overall improvement in host health, including bone health [15]. Studies conducted with *Limosilactobacillus reuteri* ATCC PTA 6475 have demonstrated that this specific strain suppresses the expression of pro-inflammatory and pro-osteoclastogenic cytokines in both the intestine and bone marrow [16–18]. Furthermore, apart from the encouraging outcomes observed in animal studies, the regular intake of this strain for 12 months among older women with low bone mineral density (BMD) decreased overall tibial BMD loss [19] compared to the placebo group.

It is essential to consider the challenges associated with the product composition and storage conditions when dealing with nutrition supplements or drugs. To be classified as probiotics, microorganisms must be alive, and the viability of products containing probiotics can be affected by variables such as temperature, moisture and oxygen [20]. Although probiotics are generally considered safe for healthy adults, using live bacteria has been linked to an increased risk of infection or complications in specific populations. This includes young infants and neonates, critically ill adult and infant patients in intensive care units, and postoperative, hospitalized, or immunocompromised patients. The risks are partially attributed to the occurrence of bacteremia and fungemia [21, 22]. We must emphasize that, as far as we know, there have been no reports of any adverse effects in healthy individuals. This concern has led to a growing interest in the use of dead or non-viable bacteria [23].

The term “postbiotic” refers to using inactivated microorganisms or their components in adequate amounts to promote health benefits [24]. Postbiotics offer advantages such as easier standardization, storage, and transport [23] compared to live cells. Despite the growing interest in postbiotics, there is a limited number of studies investigating their effects on bone health [25–29], and none of them assessed the effects of heat-killed *L*. *reuteri* 6475. However, these studies have shown promising results, as the administration of postbiotics has been associated with reduced bone loss, lower bone resorption markers, increased bone density and improved bone microarchitecture.

The potential to prevent postmenopausal bone loss through a natural, non-invasive, and non-stressful method holds significant appeal. Using heat-killed *L*. *reuteri* is interesting because it would offer fewer potential adverse effects, easier production, and a product with improved stability and longer shelf-life. Considering the demand for new therapeutic alternatives or adjuncts in osteoporosis treatment and the growing use of probiotics and postbiotics, we hypothesize that heat-killed form of *L*. *reuteri* could be so effective as the viable form in preventing postmenopausal bone loss.

## Materials and methods

**Fig 1:**
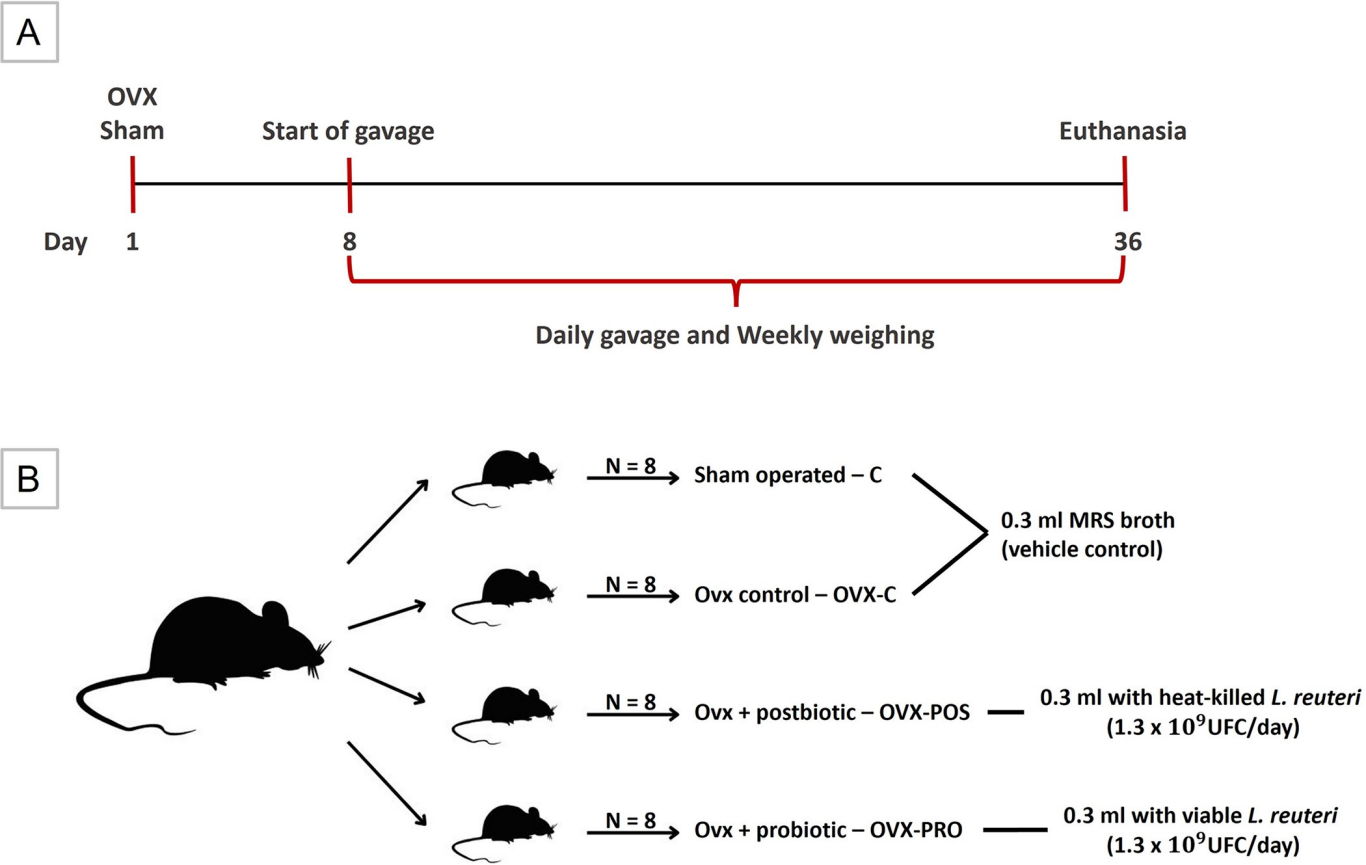
Experimental design. A) Experiment timeline, B) Experimental groups.

This is a randomized, controlled, and masked experimental study. All experimental procedures were approved by the Animal Ethics Committee of the Institute of Science and Technology of São José dos Campos (protocol 18/2019) and followed the ARRIVE guidelines. Thirty-two 18-week-old specific pathogen-free female mice (*Mus musculus*, BALB/c) were housed in a temperature- and light-controlled environment (22°C; light/dark cycle of 12/12h) and received water ad libitum. They were randomly divided into four groups (Fig 1): 1) Sham-Control group (C): *Sham-*operated mice; 2) OVX-Control group (OVX-C): Ovx mice; 3) OVX-Postbiotic group (OVX-POS): Ovx mice that received heat-killed *L*. *reuteri*; and 4) OVX-Probiotic (OVX-PRO): Ovx mice that received viable *L*. *reuteri*. Mice were treated by gavage with 0.3 ml (1.3x10^9^ CFU/day) [16] with *L*. *reuteri* and/or De Man Rogosa Sharpe (MRS) broth (dipotassium hydrogen phosphate, 2 g/L glucose, 20 g/L magnesium sulfate monohydrate, 0.2 g/L manganous sulfate tetrahydrate, 0.05 g/L meat extract, 8 g/L peptone, 10 g/L sodium acetate, 5 g/L triammonium citrate, 2 g/L yeast extract, 4 g/L; vehicle of pro- and postbiotic suspension).

Power analysis showed that a sample of 8 mice per group has 80% power to detect an effect size of 5.13 or a 22% change, assuming a significance level of 5% and a two-sided test [30].

The animals received standardized AIN-93-M chow. We determined the average food intake by weighing the remaining food in each cage three times a week for two weeks before the Ovx. Following the surgery, the animals were provided with only the average amount of food intake to prevent weight gain from the Ovx. Body weight measurements were taken on the day of surgery and weekly thereafter until the day of euthanasia.

### Ovx and sham surgery

After a two-week acclimatization period, all animals, previously weighed, were anesthetized with isoflurane (2% for induction and 1.8% for maintenance; oxygen 2 L/min) and submitted to Ovx or sham surgery on day 1 (Fig 1). Bilateral Ovx was performed through a bilateral longitudinal skin incision in the lateral abdominal region, below the last rib, and total excision of the ovaries, after ligation of the uterus with absorbable suture (4–0, Shalon Ltda). Sham surgery was done by exposing the ovaries without excision. The peritoneal muscle was sutured with an absorbable suture thread, and skin with a silk thread (4–0, Ethicon, Johnson & Johnson). The animals received analgesic and anti-inflammatory medication subcutaneously in the three days following surgery (meloxicam: 2 mg/kg; tramadol: 5 mg/Kg). The efficacy of Ovx was confirmed during euthanasia by uterine atrophy.

### Bacterial strains and culture

One week after the Ovx/sham surgery, the administration of probiotic, postbiotic or MRS broth was started (Fig 1). *L*. *reuteri* ATCC PTA 6475 (BioGaia AB) was cultured under anaerobic conditions in MRS broth (Difco) at 37°C. Single colonies were confirmed by Gram stain from the agar. After 24 hours of growth, cells were harvested by centrifugation at 5,000 rpm for 10 minutes, the supernatant was discarded, the bacterial pellet was resuspended in MRS broth, and the standardization of the microorganism was performed in a spectrophotometer with 600 nm wavelength (Optical density 1 = 2.34 x 10^9^ CFU/ml) [16]. For postbiotic preparation, the suspension with live microorganisms was autoclaved at 121°C for 20 minutes and then plated on MRS agar to ensure no viable cells remained. The postbiotic was prepared in sufficient quantity once per week and stored in a refrigerator. Probiotics and postbiotics were administered to the mice in MRS broth.

### Euthanasia

Euthanasia was performed after four weeks of treatment (on day 36–35 days after the day of Ovx or sham surgery) (Fig 1). Animals were anesthetized using intraperitoneal overdose injection of ketamine and xylazine followed by decapitation. The uterus was weighed immediately to assess atrophy after Ovx. The right femur was fixed in paraformaldehyde 4% (phosphate buffer pH 7.4; 0.1 M). The ileum was immediately frozen in liquid nitrogen and stored in a -80°C freezer. An external examiner coded the samples, and the researchers were blinded before data evaluation.

### Scanning electron microscopy (SEM)

Probiotic and postbiotic samples were analyzed by scanning electron microscopy (SEM) to visualize bacterial structural alterations after heat inactivation. The SEM (JEOL JSM-7900 F Scanning Electron Microscope, JEOL USA, Inc.) procedures were performed according to Garcia et al. [31] with some modifications. Collected from the culture in MRS broth (1 μl), the bacteria were placed in a small pre-delimited area of the Petri dish and fixed in 2% glutaraldehyde for 1 h. The samples were dehydrated in increasing ethanol concentrations (10, 25, 50, 75 and 90%) for 20 min each, followed by 1 h immersion in 100% alcohol. The samples were then incubated at 37°C for 24 h for total drying and transferred to aluminum stubs and sputter coated with gold for 160 s at 40 mA (Denton Vacuum Desk II).

### Micro-computed tomography (μ-CT)

Three-dimensional μ-CT analyses of the distal femoral metaphysis were performed using SkyScan (SkyScan 1176 Bruker MicroCT). Tomographic images were acquired at 50 kV and 500 μA with an isotropic voxel size of 17.48 μm; aluminum filter of 0.5 mm, and an integration time of 380 ms. The images were reconstructed three-dimensionally, with the aid of the NRecon (SkyScan, Version 1.6.6.0) and Data Viewer (SkyScan, Version 1.4.4 64-bit) software, and a volume of interest (VOI) was standardized from a transaxial section, using the CT Analyzer software (CTAn-2003-11, SkyScan, Version 1.12.4.0). The analyzed VOI corresponded to 100 CT scans of the trabecular bone at the femoral metaphysis located 25 cuts away from the most proximal point of the growth plate. Bone volume fraction (BV/TV), trabecular number (Tb.N), thickness (Tb.Th), separation (Tb.Sp), total porosity (Po.tot) and structural model index (SMI) were calculated using standard methods.

### Histology

For the descriptive histologic evaluation, the femurs were decalcified in 0.5M ethylenediaminetetraacetic acid (EDTA) solution (pH 7.8) and embedded in paraffin. Five-micrometer-thick sections were cut and stained with hematoxylin and eosin (H&E).

### RT-qPCR

The tissues were pulverized using a liquid N_2_ cooled mortar and pestle and then transferred to TRIzol (Invitrogen). RNA was extracted, and the quality was confirmed by the presence of rRNA bands on agarose gel electrophoresis. After treatment with DNase (RQ1 RNase-Free DNase, Promega), 1 μg of RNA was transcribed into cDNA using the GoScript^TM^ Reverse Transcriptase Kit (Promega), and all protocols were performed as recommended by the manufacturers. After analyzing the *Gapdh*, *Tbp* and *Hprt* genes, *Gapdh* was the endogenous control of choice. The primers were designed, and the sequences are summarized in Table 1. The specificity of the reaction was checked by melting curve analysis and amplification efficiency. Evaluation of gene expression was performed using the 2^-ΔΔCT^ method.

**Table 1:** Primer sequences for RT-qPCR reaction.

Gene	Forward	Reverse	Amplicon size
*Gapdh*	AGGTCGGTGTGAACGGATTTG	TGTAGACCATGTAGTTGAGGTCA	123
*Tbp*	CCTTGTACCCTTCACCAATGAC	ACAGCCAAGATTCACGGTAGA	119
*Hprt*	TCAGTCAACGGGGGACATAAA	GGGGCTGTACTGCTTAACCAG	142
*Ocl*	CCTTCTGCTTCATCGCTTCC	AGCGCTGACTATGATCACGA	104
*Il-6*	TAGTCCTTCCTACCCCAATTTCC	TTGGTCCTTAGCCACTCCTTC	76
*Tnf-α*	CCCTCACACTCAGATCATCTTCT	GCTACGACGTGGGCTACAG	61

### Statistical analysis

The statistical analysis was performed in two stages–in the first, the Ovx groups (OVX-Control, OVX-Postbiotic and OVX-Probiotic) were compared with the Sham-Control group; and in the second, the OVX groups were compared to each other. Data were evaluated using the analysis of variance (ANOVA) or non-parametric Kruskal-Wallis’ test (α = 0.05). The Dunnett, Dunn and Tukey posthoc tests were used in case of statistical difference. Tests were performed using GraphPad Prism version 6.01 (GraphPad Software, Inc).

## Results

The body weight (g) on day 1 and day 36 was compared. At both time points, no statistically significant difference was observed among the groups, confirming the effectiveness of the feeding control (p = 0.2527, Kruskal-Wallis and p = 0.4188, ANOVA; day 1 and day 29, respectively). Bilateral Ovx leads to uterine atrophy, confirmed by weighing the uterus at the time of euthanasia [32]. When compared to the control, the Ovx groups had lower uterine weight (g), with a statistically significant difference (p = 0.0004, Kruskal-Wallis, Dunn).

### Effects of heat inactivation on cell morphology

**Fig 2:**
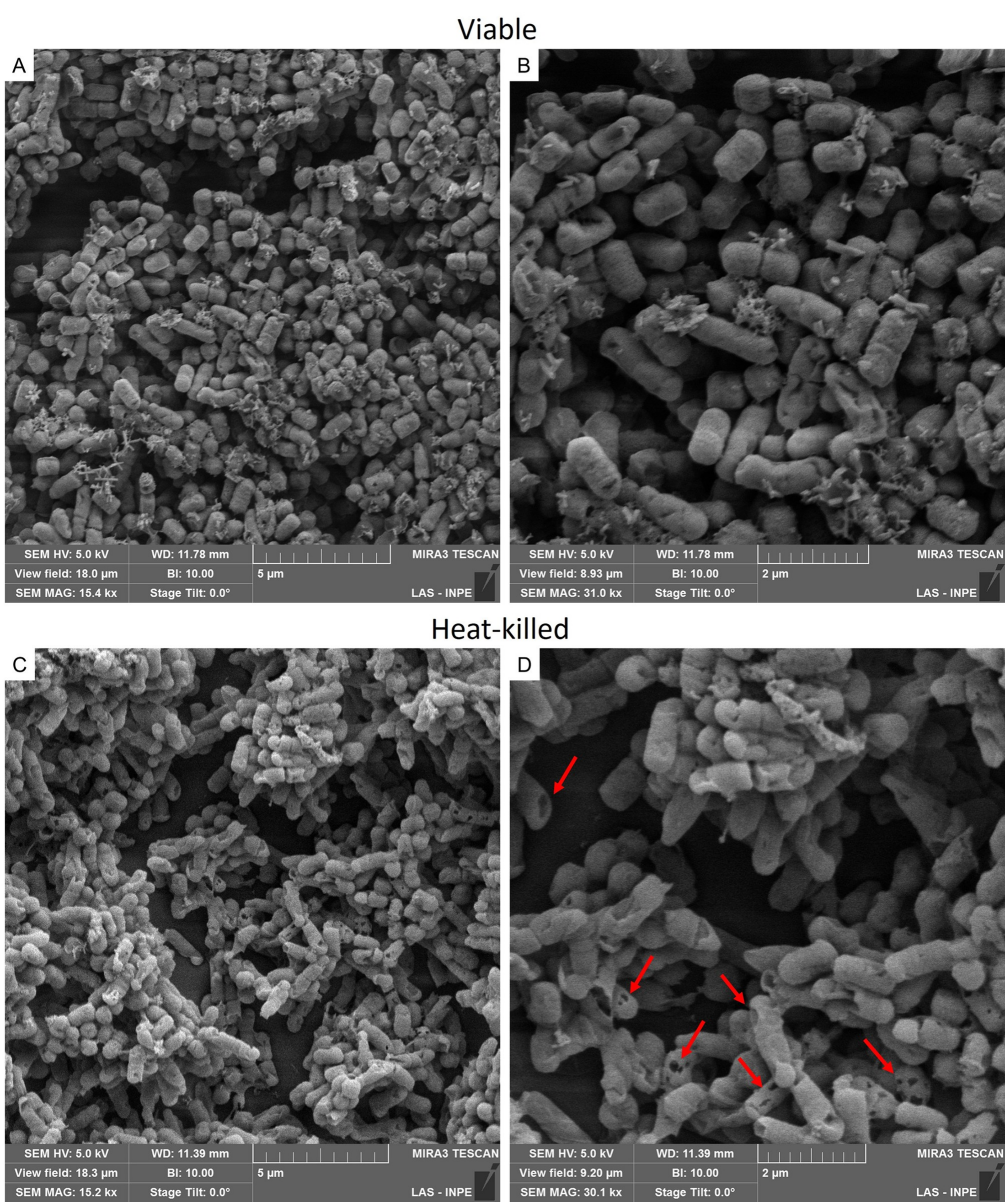
Scanning electron microscopy images of *L*. *reuteri*. *L*. *reuteri* formed characteristic chain arrangement. The probiotic (A and B) presented a short rod shape, without changes on the surface, while the heat-killed form (C and D) showed some punctual disruptions on the cell wall (red arrows). Figures A and C were taken at 15,000x magnification, and B and D at 30,000x magnification.

The images acquired through SEM confirmed the structural integrity of the probiotic (Fig 2A and 2B), as well as the preservation of the heat-killed *L*. *reuteri*’s structure, which remained largely intact (Fig 2C). Only a few disruptions were observed on the cell surface (Fig 2D), resembling the appearance of the live probiotic.

### Bone microarchitecture and histology

**Fig 3:**
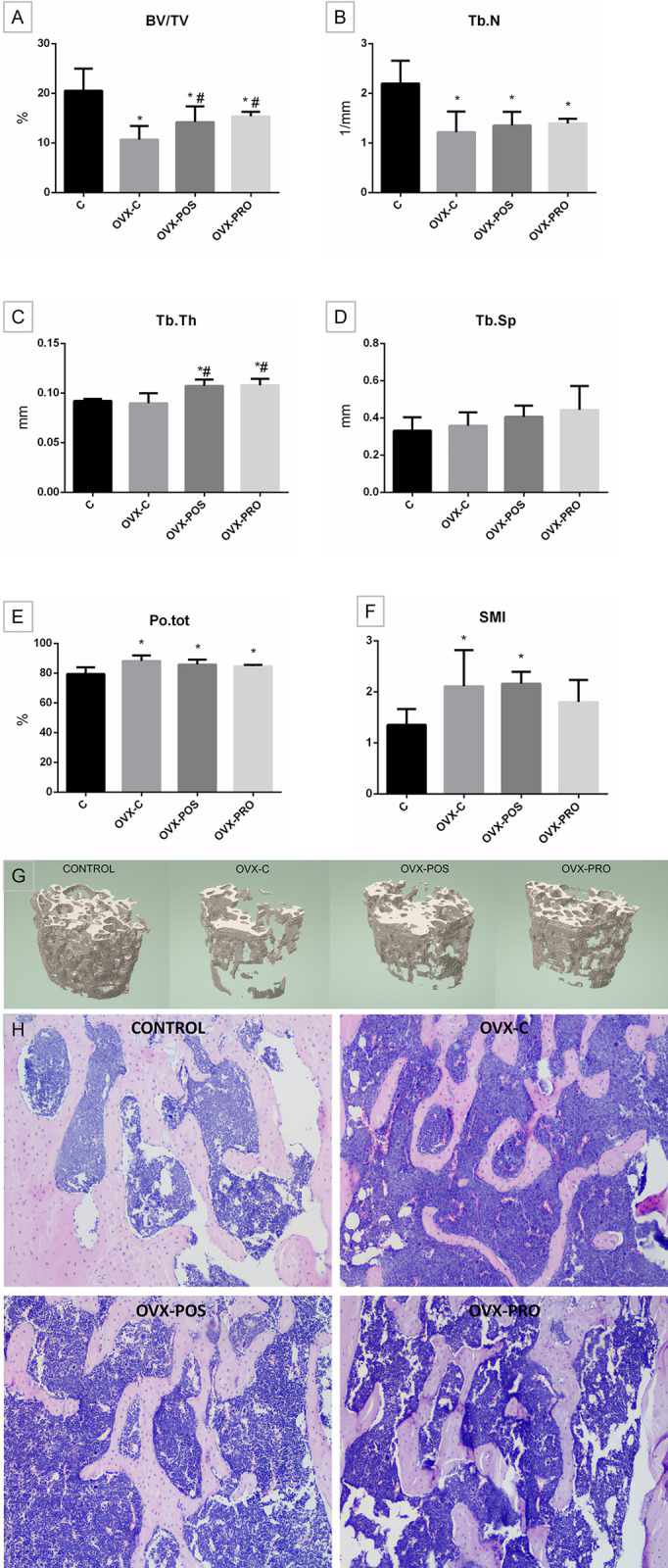
Bone microarchitecture and histology. Graphs of means ± standard deviation of A) bone volume fraction (BV/TV–%), B) trabecular number (Tb.N– 1/mm), C) trabecular thickness (Tb.Th–mm), D) trabecular separation (Tb.Sp–mm), E) total porosity (Po.tot–%), F) structure model index (SMI); G) three-dimensional reconstruction of the volume of interest reveals that Ovx led to increased bone loss, while both treatments effectively mitigated it, and H) histological analysis.* p < 0.05 when compared to C; # p < 0.05 when compared to OVX-C (n = 8).

A statistically lower BV/TV (p < 0.0001, ANOVA, Dunnett, Fig 3A), Tb.N (p < 0.0001, ANOVA, Dunnett, Fig 3B) and greater Po.tot (p = 0.0004, ANOVA, Dunnett, Fig 3E) were found in the OVX groups when compared to the C group.

In addition, the OVX-POS and OVX-PRO groups showed a positive and similar effect of treatment with heat-killed and viable *L*. *reuteri*, respectively, with a higher BV/TV when compared to OVX-C (p = 0.0077, ANOVA, Tukey, Fig 3A). Treatment with both forms of *L*. *reuteri* had a positive and similar effect on Tb.Th, where both groups showed greater Tb.Th when compared to C (p < 0.0001, ANOVA, Dunnett) and OVX-C (p = 0.0002, ANOVA, Tukey, Fig 3C). In the Tb.Sp analysis, groups were statistically similar (p = 0.0762, ANOVA, Fig 3D). The SMI was significantly increased for OVX-C and OVX-POS compared to C (p = 0.0053, ANOVA, Dunnett), while OVX-PRO showed no significant alterations.

The morphological results after evaluation of decalcified sections are similar to microtomography findings (Fig 3H).

### *Occludin*, *Il-6*, and *Tnf-α* gene expression in the intestine

**Fig 4:**
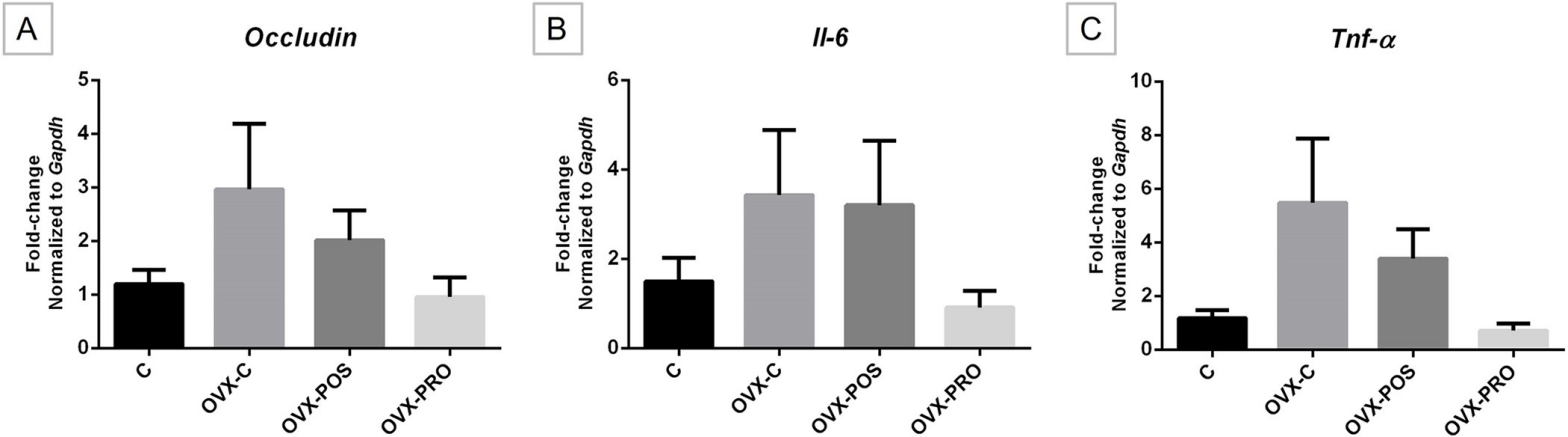
Graphs of means ± standard error of gene expression. A) *Occludin* B) *Il-6*, and C) *Tnf-α* mRNA expression in the ileum, showing similar expression levels among groups (C n = 7, OVX-C n = 7, OVX-POS n = 7, OVX-PRO n = 6).

In the analysis of *Occludin*, *Il-6* and *Tnf-α* gene expression, the Ovx groups maintained statistically similar levels to the control (p = 0.1909, ANOVA; p = 0.6508, Kruskal-Wallis; p = 0.0693, Kruskal-Wallis; respectively, Fig 4). However, a trend to increased expression could be seen due to Ovx (OVX-C), as well a trend to reduction by the treatments.

## Discussion

Given the status of osteoporosis as a significant public health concern, it is crucial to explore novel and effective therapies. Probiotics and postbiotics have gained widespread attention for their potential benefits to the host. Studies have demonstrated the positive impact of probiotics [33–37] and postbiotics [25–29] on bone health. In the present study, we investigated the effects of *L*. *reuteri* ATCC PTA 6475, both in its viable and heat-killed forms, on the maintenance of bone volume after Ovx in mice.

During menopause, there is a decline in ovarian hormone production, which can result in hyperphagia [38], increasing body weight and adipose tissue mass [39]. Elevated body mass index (BMI) is correlated with increased bone density [40], which can be explained by mechanical and hormonal mechanisms. Bone cells can detect heightened mechanical load, and, in response, increase bone formation [41]. Furthermore, estrogen can be synthesized from adipose tissue, which, in addition to greater mechanical stress exerted, may also play a role in elevating BMD [42]. We implemented strict animal feeding control to prevent weight gain resulting from Ovx and its potential impact on bone mass. Throughout the experiment, the animals’ weight remained relatively stable, indicating the effectiveness of the feeding control measures. Furthermore, immediate weighing of the uterus after euthanasia revealed uterine atrophy in the OVX groups, confirming the success of the Ovx procedure.

Emerging evidence suggests that probiotics and gut microbiota play a crucial role in regulating bone health [7]. In healthy male mice, the administration of *L*. *reuteri* has been shown to reduce intestinal inflammation, enhance osteoblast activity, and promote increased bone formation [18]. In a previous study, ovariectomized mice treated with *L*. *reuteri* ATCC PTA 6475 demonstrated a decrease in bone loss [16], which was also observed in this study since the treatment with *L*. *reuteri* 6475 protected mice from estrogen deficiency-induced bone loss by improving BV/TV and Tb.Th. Viable *L*. *reuteri* 6475 can be detected up to 5 days in fecal samples after the administration of 10^9^ cells for two consecutive days to mice [43], suggesting that the aforementioned benefits are related to viable cells that riches the intestine. Furthermore, confirming our hypothesis, and in accordance with other authors, the postbiotic also positively affected bone health [25–29]. We found that the postbiotic had similar effect as the probiotic on the bone microarchitecture.

Postbiotic preparations encompass intact cells or structural fragments, such as cell walls, bacterial lysates, cell-free supernatants, metabolites, and proteins, all contributing to beneficial health effects [24]. The specific inactivation method can influence the postbiotic effects as bacterial cells can be disrupted, releasing compounds that interact with host cells [44]. Thermal treatment, involving heat application for a specified duration, is the most common method used for inactivation [45]. Our study used sterilization (> 100°C), which resulted in minimal disruptions on the bacterial surface while maintaining the anticipated bone effects. The mechanisms underlying the health benefits of postbiotics are not fully understood but involve immunomodulatory effects [46–50] and antimicrobial properties [51]. Similar to probiotics, postbiotics can adhere to and counteract pathogens [51]. Inactivated cells are recognized by antigen-presenting cells, triggering the production of anti-inflammatory cytokines while suppressing the production of pro-inflammatory cytokines [49, 50]. Additionally, probiotics exert their effects through the production of metabolites (such as short-chain fatty acids) [5] and vitamins [52], enhanced mineral absorption [53], and the upregulation of TJs [10]. Based on our findings, the impact of probiotics on bone microarchitecture is not dependent on its viability.

To elucidate a portion of the mechanism underlying the effects of pro- and postbiotic on bone, we investigated the gene expression of a protein associated with intestinal permeability and two pro-inflammatory cytokines. TJs play a crucial role in maintaining the integrity and function of the intestinal barrier [54], and sex steroid deficiency increases gut permeability and upregulates osteoclastogenic cytokines through an inflammatory response [55]. Occludin plays an important role in paracellular diffusion [56]. Our study did not find a statistically significant difference in *Occludin* gene expression between the groups. Collins et al. (2017) [57] have reported that estrogen deficiency resulting from Ovx induces region- and time-dependent effects on gut TJs. One limitation of our study is that we only assessed *Occludin* in the ileum at a single time point, while multiple families of proteins are involved in TJs.

Estrogen deficiency has been associated with an increase in the expression of pro-inflammatory cytokines [57], and previous studies have demonstrated the effects of probiotics on cytokine expression [16–18]. TNF-α, a pro-inflammatory cytokine, has been shown to disrupt TJ proteins and increase intestinal permeability [58]. In our study, although not statistically significant, there was a trend towards upregulation of *Tnf-α* and *Il-6* gene expression in the Ovx groups. Collins et al. (2017) [57] found that increased gene expression of *Tnf-α* in the ileum was correlated with upregulated expression of *Occludin*. Also, the compensatory increase in *Occludin* gene expression is believed to play a direct role in enhancing the epithelial barrier function [59]. Moreover, the graphs displayed a coherent pattern, indicating a tendency towards decreased *Ocluddin* and cytokine’s expression in the groups that received *L*. *reuteri* in its viable and heat-killed form.

The utilization of heat-killed *L*. *reuteri* has yielded favorable outcomes as a therapeutic choice, affirming that live bacteria aren’t always necessary for beneficial effects. Ensuring probiotics’ viability in products is crucial, making postbiotics highly financially attractive due to their extended shelf life, stability across various pH and temperature ranges, and the ability to be incorporated into foods before thermal processing without losing functionality [60]. It’s imperative to understand the long-term stability and efficacy of these products for their successful integration into clinical settings.

The use of postbiotics presents a significant opportunity for new therapy development, particularly for at-risk populations. The promising outcomes from using *L*. *reuteri*, whether viable or inactivated, underscore its potential applications in preventing and treating postmenopausal bone loss. This paves the way for further research with both theoretical and practical implications. Future investigations should prioritize high-quality, standardized randomized clinical trials to elucidate the specific mechanisms of action for both forms of *L*. *reuteri*.

In conclusion, our study provides evidence that the beneficial effects of *L*. *reuteri* ATCC PTA 6475 in the maintenance of bone volume in ovariectomized mice are not contingent upon cell viability. Our findings suggest that viable and heat-killed *L*. *reuteri* ATCC PTA 6475 had similar beneficial effects on maintaining the bone volume percentage independently of gut *Occludin* and intestinal *Il-6* and *Tnf-α* gene expression. However, the precise mechanisms of action remain unclear, and further investigations are warranted to elucidate them.

## Supporting information

S1 Table Femur bone parameters. (DOCX)
