# Supplementary material for: Heat-killed Limosilactobacillus reuteri ATCC PTA 6475 prevents bone loss in ovariectomized mice: A preliminary study
Source: PLoS One. 2024 May 31;19(5):e0304358. doi: 10.1371/journal.pone.0304358 (PMC11142514; doi:10.1371/journal.pone.0304358)
Supplement: S1 Table — (DOCX) [file pone.0304358.s001.docx]

**S1 Table. Femur Bone Parameters**

|  | **Control** | **OVX-C** | **OVX-POS** | **OVX-PRO** |
| --- | --- | --- | --- | --- |
| **BV/TV (%)** | 20.51 ± 4.48 | 10.66 ± 2.75* | 14.18 ± 3.20*^#^ | 15.24 ± 0.93*^#^ |
| **Tb.Th (mm)** | 0.092 ± 0.001 | 0.089 ± 0.010 | 0.107 ± 0.006*^#^ | 0.107 ± 0.006*^#^ |
| **Tb.N (1/mm)** | 2.19 ± 0.46 | 1.21 ± 0.41* | 1.35 ± 0.27* | 1.39 ± 0.09* |
| **Tb.Sp (mm)** | 0.33 ± 0.07 | 0.35 ± 0.07 | 0.40 ± 0.05 | 0.44 ± 0.12* |
| **Po.tot (%)** | 79.49 ± 4.48 | 88.09 ± 3.76* | 85.82 ± 3.20* | 84.66 ± 0.93* |
| **SMI** | 1.35 ± 0.31 | 2.10 ± 0.70* | 2.15 ± 0.23* | 1.79 ± 0.43 |

Femur distal trabecular bone parameters. Control, sham-mice; OVX-C, ovariectomized; OVX+POS, Ovx mice treated with heat-killed *L. reuteri*; OVX+PRO, Ovx mice treated with viable *L. reuteri*. BV/TV, bone volume fraction; Tb. Th, trabecular thickness; Tb. N, trabecular number; Tb. Sp, trabecular separation; Po.tot, total porosity; SMI, structure model index. Data are means ± standard deviation, n=8 per group.

**p*<0.05 to Control

*#p*<0.05 to Ovx-C by one-way ANOVA followed by Tukey HSD.
